# Breastfeeding in Pediatric Acute-Onset Neuropsychiatric Syndrome: An Italian Observational Study

**DOI:** 10.3389/fped.2021.682108

**Published:** 2021-07-08

**Authors:** Manuel Murciano, Davide Maria Biancone, Francesca De Luca, Denise Piras Marafon, Cristiana Alessia Guido, Alberto Spalice

**Affiliations:** ^1^Emergency Paediatric Department, Bambino Gesù Children's Hospital, Rome, Italy; ^2^Child Neurology Division, Department of Pediatrics, “Sapienza” University of Rome, Rome, Italy; ^3^Rheumatology Unit, Bambino Gesù Children's Hospital, Rome, Italy; ^4^Department of Developmental and Social Psychology, Faculty of Medicine and Psychology, Sapienza University of Rome, Rome, Italy

**Keywords:** pediatric acute-onset neuropsychiatric syndrome, pediatric autoimmune neuropsychiatric disorders associated with streptococcal infection, breast feeding, neurodevelopment, milk, infant formula, children

## Abstract

**Objective:** Pediatric acute-onset neuropsychiatric syndrome (PANS) is a condition defined by sudden onset of obsessive–compulsive symptoms and/or severe eating restrictions, along with at least two other cognitive, behavioral, or neurological symptoms. Its pathogenesis is unknown but it seems triggered by infections, metabolic disturbances, and other inflammatory reactions. PANS represents a neurodevelopmental problem and infant feeding can play a role. Breast milk is the ideal food for infants and influences children's brain, cognitive, and socio-emotional development.

**Methods:** We enrolled 52 children diagnosed with PANS. We interviewed their parents in order to investigate perinatal history, infant feeding, neurologic development, and confounding factors like socio-economic status and region of origin. We subgrouped PANS patients into three subsets: those who only received human milk (HMO), those who only received infant formula, and those who received mixed feeding.

**Results:** The cohort is composed of 78.9% males, with a median age of 11 years (range 7–17). We found some neurodevelopmental problems (13.5%): walking disorders, ASD, ADHD, oppositional attitude, and delayed psychomotor development. We found scholar performance deficits (25%), including language problems like dysgraphia, dyslexia, and dyscalculia. The achievement of some milestones in the development of the infant is affected in 73.1% of cases. Breastfeeding is not homogeneously practiced in Italy because of social, economic, and cultural phenomena. The richest and the poorest families (100%) in the sample choose breastfeeding, probably with a different approach and for different reasons (awareness or need). In the group of PANS patients fed with HMO, compared to the rest of the patients, we registered fewer cases of growth problems (0 vs. 12.9%; *p* = 0.14), school performance problems or the need for school support (19.1% vs. 29%; *p* = 0.42), and a delay in the age of babbling/speaking (range 4–20 vs. 7–36 months; *p* = 0.066).

**Conclusion:** This is the first study that investigates the role of breastfeeding in the development of PANS. Promoting breastfeeding is important in the general population and also in PANS patients because it has an important social and global health impact, also during adult life. Further studies with a bigger population are needed to investigate the mechanisms underlying PANS and the role that breastfeeding may play in their short- and long-term neurodevelopment.

## Article Summary

This study investigates the role that breastfeeding may play in short- and long-term neurodevelopment in PANS patients, from birth to 15 years.

## What's Known on This Subject

PANS is a condition defined by sudden onset of obsessive–compulsive symptoms and/or severe eating restrictions, along with at least two other cognitive, behavioral, or neurological symptoms. The pathogenesis of PANS is unknown. Breast milk influences children's brain, cognitive, and socio-emotional development.

## What This Study Adds

This study describes the neurodevelopmental evolution and problems among 52 PANS patients from intra-uterine life till adolescence and focuses on infant feeding, human milk, and formula, looking for a correlation with neurologic infant milestones and scholar outcomes.

## Introduction

### Pans and Pandas

**Figure 1:**
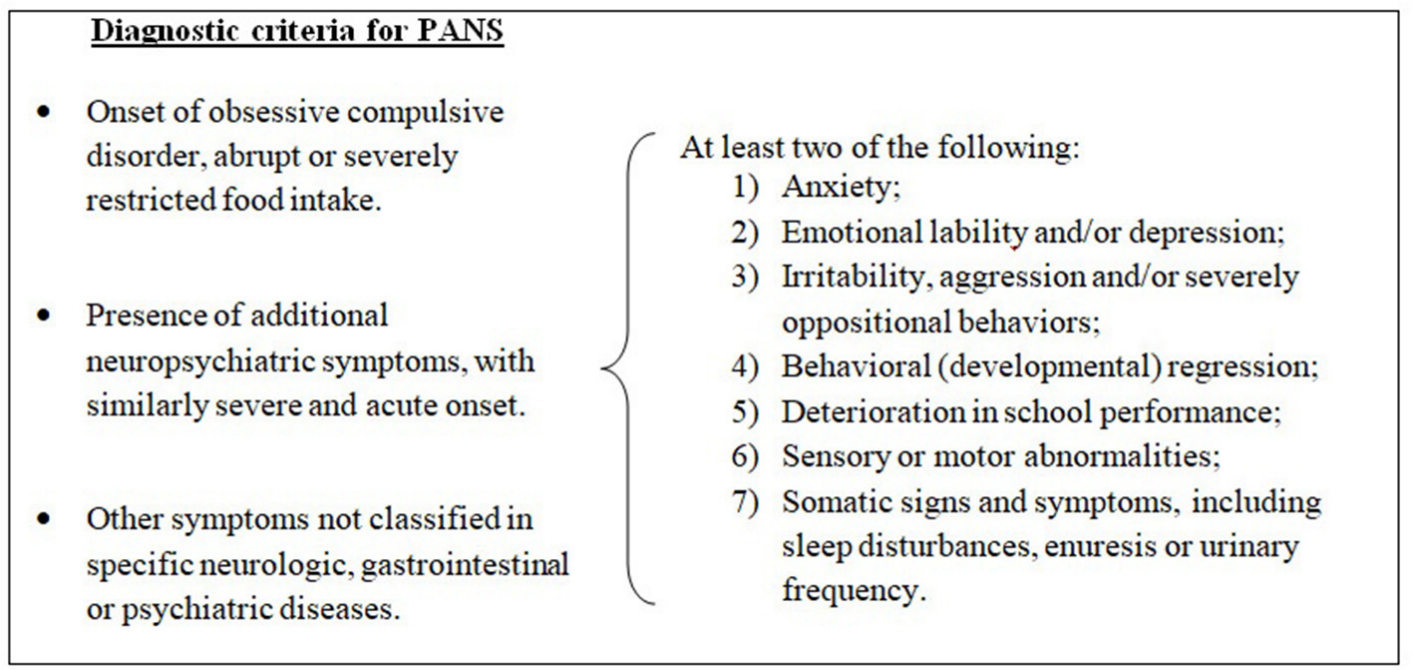
Diagnostic criteria for pediatric acute-onset neuropsychiatric syndrome (PANS), modified from 2013 PANS Consensus Conference (2, 3).

Pediatric acute-onset neuropsychiatric syndrome (PANS) is a condition defined by sudden onset of obsessive–compulsive symptoms and/or severe eating restrictions, along with at least two other cognitive, behavioral, or neurological symptoms (1) (see Figure 1).

**Figure 2:**
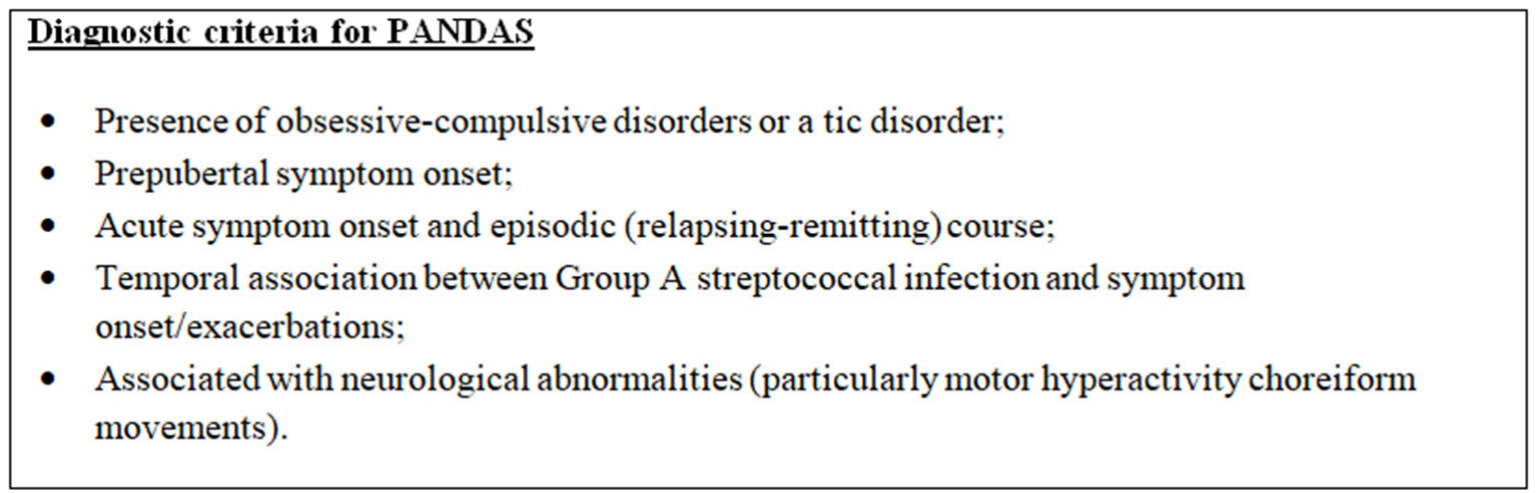
Diagnostic criteria for Autoimmune Neuropsychiatric Disorder Associated with Streptococcal Infections (PANDAS), modified from 2013 PANS Consensus Conference (2, 3).

The pathogenesis of PANS is unknown, but it seems triggered by infections, metabolic disturbances, and other inflammatory reactions. Children diagnosed with PANDAS (Pediatric Autoimmune Neuropsychiatric Disorder Associated with Streptococcal Infections) have an acute onset of neuropsychiatric symptoms, specifically obsessive–compulsive disorders (OCDs) or tics. PANDAS is classified as a subset of PANS (4, 5) (see Figure 2).

In 2012, PANS clinical criteria were described (6) and then updated during the 2013 PANS Consensus Conference (2, 3). The PANS definition no longer includes tic disorders as a primary criterion or the restriction of cases to prepubescent children, but it emphasizes OCD and restrictive eating. This classification is helpful to standardize cohorts of patients presenting typical symptoms (2, 3, 6). The inclusion of abrupt onset of psychiatric symptoms as a criterion seems to distinguish a subset of children from others referred for evaluation for PANS.

PANS describes the clinical presentation of a subcategory of childhood OCD. PANS or PANDAS syndrome should be considered whenever symptoms of OCD, food restrictions, or tics occur and are accompanied by other emotional and behavioral changes, frequent urination, motor abnormalities, and/or changes in writing. Some authors in the literature have also correlated the close connection of the pathology with ENT (ears, nose, and throat) symptoms and the reduction of behavioral disorders after medical or surgical treatment (7–9).

Even if the pathogenesis of PANS is not clear, several studies hypothesize a mechanism known as “molecular mimicry” or rather the immunological cross-reactivity between host and bacterial antigens, above all regarding the PANDAS subgroup (10–15). It is known that the lymphocytic responses against microbial pathogens can be auto-reactive to the basal ganglia and the surrounding brain tissues (16, 17). Many PANS patients have concurrent autoimmune/inflammatory diseases: the most common are arthritis and thyroiditis. These patients have higher levels of antinuclear, anti-histone, and anti-thyroid antibodies than the general population (18). Symptoms appear to improve administering anti-inflammatory and immunosuppressive therapies (19, 20). In allergic diseases, there is a Th2-mediated immunological response (21), but the involvement of this pathway in PANS pathogenesis is not clear.

### Breastfeeding, Infant Formula, and Neurodevelopment

There are no previous studies known to the authors that have investigated the role of breastfeeding in the development of PANS. Breast milk is the ideal food for infants (22, 23). It is safe and clean and contains antibodies that help protect the infant against many common childhood illnesses (24–28). Breast milk influences children's brain, cognitive, and socio-emotional development (29). WHO (World Health Organization) and UNICEF (United Nations International Children's Emergency Fund) recommend to mothers to start breastfeeding in the first hour of life and to continue to do so exclusively for the first 6 months. It is essential for the survival, growth, and development of the baby in the first year of life, with an impact that affects adult life (30).

When exclusive breastfeeding is insufficient or may not be available, suitable, or solely adequate, additional milk intake should be provided. The choice of an adequate infant formula (23, 31), which mimics breast milk nutritional composition (23) (see Table 1), should be administrated considering the infant's characteristics and specific nutritional needs (32).

**Table 1:** Comparison between breast milk and infant formula on the market in Italy around 2008, in the period in which the patients in the study were infants.

**Formula 2012**	**Aptamil^®^ 1**	**Humana^®^ 1**	**Mellin^®^ 1**	**Nidina^®^ 1**	**Nipiol^®^ 1**	**Human Milk**
Kcal/dl	66	68	66	67	69	70
Proteins source	S/C 60/40	S/C 60/40	S/C 60/40	S/C 70/30	S/C 52/48	S/C 70/40
Proteins g/dl	1.3	1.7	1.3	1.2	1.4	1.2
Lipids source	Palm, canola, coconut, and sunflower oil; fish oil. DHA 6.4 mg/dl; ARA 11 mg/dl; EPA 1.4 mg/dl	Palm, rapeseed, sunflower and palm kernel oil; fish oil. DHA 7 mg/dl; ARA 7 mg/dl	Palm oil, rapeseed, coconut, sunflower; fish oil. DHA 6.4 mg/dl; ARA 6.4 mg/dl; EPA 1.4 mg/dl	Vegetable oils; fish oil. DHA 8.2 mg/dl; ARA 8.2 mg/dl	Vegetable oils (including soybean oil)	Traces of fatty acids with 8 C atoms; Polyunsaturated fatty acids 14%; DHA; ARA; EPA
Lipids g/dl	3.4	3.5	3.4	3.6	3.6	3.8
Carbohydrates source	Lactose 7 g/dl; Glucose 0.2 g/dl. GOS/FOS (9/1) 0.8 g/dl	Lactose 6.7 g/dl; Maltodextrins 0.2 g/dl; Glucose 0.2 g/dl. GOS 0.5 g/dl	Lactose 7 g/dl; Glucose 0.2 g/dl. GOS/FOS (9/1) 0.8 g/dl	Lactose 7.5 g/dl	Lactose 5.4 g/dl; Maltodextrins 2.3 g/dl	Lactose 7 mg/dl; Oligosaccharides 0.5 mg/dl
Carbohydrates g/dl	7.4	7.1	7.4	7.5	7.7	7.0
Sodium mg/dl	17	19	17	25	20	15
Potassium mg/dl	45	68	65	77	60	55
Calcium mg/dl	47	65	47	39	65	30
Phosphorus mg/dl	26	44	26	23	45	14
Nucleotides mg/dl	3.2	-	3.2	2	-	7
Osmolarity mOsm/l	300	293	-	275	n.d.	79

*S/C, Sieroproteins/Caseins ratio; GOS, galacto-oligosaccharides; FOS, fructo-oligosaccharides*.

Breast milk contains essential nutrients for neurological and cognitive development (33, 34). LC-PUFAs (long-chain polyunsaturated fatty acid) (15% of human milk fats) represent fundamental factors for the development of the central nervous system and the retina. Brain matter is made up of 60% lipids, and its composition can be influenced by diet (35–42). Breastfed babies have a higher concentration of DHA (docosahexanoic acid) in the brain than babies who receive food without LC-PUFA supplementation (43, 44).

Positive links between breastfeeding and cognitive development are clear, with long-term effects until adulthood (45–49), particularly in premature babies (50). Exclusive breastfeeding can increase the growth of white matter in the brain from 20 to 30% (51).

Six-year-old children who had received infant formula added in LC-PUFA in the first 4 months of life or had been breastfed showed reactions significantly in the association test (52, 53). They were also more efficient at understanding and solving problems in other intelligence tests and can be very significant for their learning abilities in the school setting.

The children of mothers who took more DHA during breastfeeding presented a better understanding of speeches at the age of three and better verbal and non-verbal intelligence at the age of seven (54). Breastfed babies from mothers who ate high-fat fish weekly showed better visual and motor skills at the age of 3 years. Another study found associations between breastfeeding and polymorphisms of the FADS2 gene (involved in fatty acid metabolism), increased attention span at the age of 12, less hyperactive behavior at the age of three, and a trend toward higher IQ (42).

Several factors influence the availability of breastfeeding in Italy (55, 56). The most common reasons of exclusive breastfeeding discontinuation include the perception of insufficient milk, misinterpretation of infant crying, returning to work or school, early introduction of solid foods, and lack of support (57).

The abrupt stop of breastfeeding leads to adverse health consequences for women, children, the community, and the environment; to a greater spending on national health systems; and to health inequalities (7, 58, 59).

Those issues assume much importance among PANS population because they can worsen morbidity and psychosocial burdens in patients who have behavioral disturbances (60, 61) and also need both pharmacological (62) and psychological therapy, especially cognitive behavioral therapy (63, 64).

## Aims

The aim of this study is to verify if a correlation exists between infant feeding and PANS age of onset. We also want to investigate if breastfeeding can be a protective factor for PANS symptoms and neurodevelopmental problems.

The immunologic system commitment in PANS (18) is known but still unclear, and it is possible that there is a connection between this syndrome and breastfeeding, which is involved in the development of children's immunologic and nervous system.

Another end point is to investigate if PANS children who received breastfeeding have better or worse neurologic impairments, compared to the ones who did not.

Family socio-economic status may affect the adherence to breastfeeding, the children's neurodevelopment, and possibly the development of PANS symptoms. So, we analyzed the family socio-economic status and perinatal health problems because both can be considered confounding factors and to investigate how they can be related to PANS.

## Materials and Methods

Since November 2019, we have enrolled 52 children diagnosed with PANS, between 7 and 17 years of age, referred to our center. We interviewed the patients' parents by telephone in order to investigate perinatal history, infant feeding, neurologic development, onset of symptoms, and confounding factors like socio-economic status and region of origin. Also, scholar achievement and other related problems were investigated.

We stratified the patients into three groups, depending on the kind of feeding they were exposed to during the first 4 months of life: group 1 receiving human milk only (HMO); group 2 receiving mixed feeding (MF), which is both human milk and infant formula; and group 3 receiving infant formula only (IFO).

Diagnosis of PANS or PANDAS was performed according to the 2013 PANS Consensus Conference (6).

Other major conditions represent exclusion criteria and have been excluded through targeted diagnostic analysis. Some of the most important conditions excluded are acute rheumatic fever (65), rheumatologic diseases, immunologic impairment, anti-phospholipids syndrome, acute or chronic infections, acute pharyngitis, encephalitis, meningitis, and presence of auto-antibodies in a blood sample. All patients had a negative culture of the pharyngeal swab at the time of the enrolment.

Most of the patients present tics and OCD symptoms spectrum, but some patients also presented other psychiatric conditions (like oppositional behaviors, selectivity for clothes or food).

We asked parents some information about family income bracket since it was found that socio-economic status can affect the development of children. We grouped all the cases into five classes of annual income bracket:

- Class 1: 10,000€−15,000€;
- Class 2: 15,000€−30,000€;
- Class 3: 30,000€−50,000€;
- Class 4: 50,000€−70,000€;
- Class 5: 70,000€−100,000€.

We found the composition of infant formula that parents reported to have been using for their children and which were on the market in Italy around 12 years ago, when most of our patients were infants. We show these data in Table 1, comparing them with breast milk.

The neurodevelopmental problems (13.5%) that we found among the 52 patients are as follows: walking disorders, autism spectrum disorder (ASD), attention deficit hyperactivity disorder (ADHD), oppositional attitude, and delayed psychomotor development. The latter, as specified in Tables 2, 3, includes the delay in the development of the infant's developmental milestones: head maintenance, sitting position, crawl, toddle/walk, and babbling. In these disorders, we have not taken into account tics and OCD, as they are typical manifestations of PANS.

**Table 2:** Characteristics of the study population and of the subgroups.

	**Total**	**Human milk only (HMO)**	**Mixed feeding (MF)**	**Infant formula only (IFO)**
	***N =* 52**	***N =* 21**	***N =* 26**	***N =* 5**
Gender, male^a^	41 (78.9)	15 (71.4)	22 (84.6)	4 (80.0)
Age at onset (years)^b^	6.0 (1.5–10.0)	5.0 (1.5–8.0)	7.0 (1.5–10.0)	5.0 (3.0–5.0)
Age (years)^b^	11 (7-17)	10 (7-17)	11 (9-17)	10 (9-12)
Region of origin^a^
Northern Italy	5 (9.6)	2 (9.5)	2 (7.7)	1 (20.0)
Central Italy	35 (67.3)	11 (52.4)	20 (76.9)	4 (80.0)
Southern Italy and Islands	12 (23.1)	8 (38.1)	4 (15.4)	0 (0.0)
Tic^a^	50 (96.2)	20 (95.2)	25 (96.2)	5 (100.0)
OCD^a^ (obsessive-compulsive disease)	25 (48.1)	12 (57.1)	11 (42.3)	2 (40.0)
Other neuro-psychiatric symptoms^a^	30 (57.7)	11 (52.4)	16 (61.5)	3 (60.0)
Gluten sensitivity or Celiac disease^a^	4 (7.7)	2 (9.5)	2 (7.7)	0 (0.0)
Father age at birth (years)^b^	36 (25–53)	36 (30–47)	36 (27–53)	36 (25–38)
Mother age at birth (years)^b^	34 (20–41)	33 (28–41)	35 (24–40)	32 (20–36)
Siblings^b^	1 (0–3)	1 (0–3)	1 (0–2)	1 (0–2)
First-born^a^	24 (46.2)	11 (52.4)	11 (42.3)	2 (40.0)
Twins^a^	4 (7.7)	0 (0.0)	3 (11.5)	1 (20.0)
Birth weight (g)^b^	3,300 (700–4,370)	3,470 (2,640–4,370)	3,190 (2,200–4,030)	3,000 (700–3,720)
Birth length (cm)^b^	50 (32–56)	50 (41–56)	50 (42–54)	50 (32–56)
Birth weight^a^
SGA (small for gestational age)	7 (13.5)	2 (9.5)	5 (19.2)	0 (0.0)
AGA (adequate for gestational age)	39 (75.0)	16 (76.2)	18 (69.2)	5 (100.0)
LGA (large for gestational age)	6 (11.5)	3 (14.3)	3 (11.5)	0 (0.0)
Apgar 1'^b^	9 (3–10)	9 (4–10)	9 (6–10)	9 (3–9)
Apgar 5'^b^	10 (1–10)	10 (7–10)	10 (8–10)	10 (1–10)
Pregnancy problems^a^	22 (42.3)	11 (52.4)	9 (34.6)	2 (40.0)
Neonatal problems^a^	17 (32.7)	7 (33.3)	7 (26.9)	3 (60.0)
Income bracket classes^a^
Class 1	4 (7.7)	2 (9.5)	2 (7.7)	0 (0.0)
Class 2	14 (26.9)	5 (23.8)	8 (30.8)	1 (20.0)
Class 3	23 (44.2)	10 (47.6)	9 (34.6)	4 (80.0)
Class 4	8 (15.4)	2 (9.5)	6 (23.1)	0 (0.0)
Class 5	3 (5.8)	2 (9.5)	1 (3.9)	0 (0.0)
Weaning age (months)^b^	6 (4–8)	6 (4–7)	6 (4–8)	6 (4–8)
Weaning problems^a^	3 (5.8)	0 (0.0)	3 (11.5)	0 (0.0)
Allergies^a^	11 (21.2)	3 (14.3)	8 (30.8)	0 (0.0)
Growth problems^a^	4 (7.7)	0 (0.0)	3 (11.5)	1 (20.0)
Neuromotor development problems^a^	38 (73.1)	16 (76.2)	18 (69.2)	4 (80.0)
- Head maintenance (months)^b^	3 (1–8)	3 (2–8)	3 (1–5)	3 (3–4)
- Sitting position (months)^b^	6 (4–10)	6 (5–10)	6 (4–9)	5 (5–7)
- Crawl (months)^b^	9 (6–20)	9 (8–11)	9 (6–20)	9 (8–15)
- Toddle/walk (months)^b^	13 (10–24)	12 (1–18)	13 (10–24)	15 (11–18)
- Babbling (months)^b^	12 (4–36)	12 (4–20)	12 (8-36)	15 (7–20)
School performance problems or support^a^	13 (25.0)	4 (19.1)	6 (23.1)	3 (60.0)

a *Number (%);*

b *Median (1°-3°quartile)*.

**Table 3:** Comparison between the subgroups of the study cohort: HMO vs. MF+IFO groups.

	**Human milk only (HMO)**	**Mixed feeding (MF) + Infant formula only (IFO)**	**HMO vs. (MF+IFO)**
	***N =* 21**	***N =* 31**	***p*-value**
Gender, male^a^	15 (71.4)	26 (83.9)	0.32
Age at onset (years)^b^	5.0 (1.5–8.0)	6.0 (1.5–10.0)	0.35
Age (years)^b^	10 (7–17)	11 (9–17)	0.24
Region of origin^a^			0.10
Northern Italy	2 (9.5)	3 (9.7)	1.0
Central Italy	11 (52.4)	24 (77.4)	0.059
Southern Italy and Islands	8 (38.1)	4 (12.9)	0.048
Tic^a^	20 (95.2)	30 (96.8)	1.0
OCD^a^ (obsessive-compulsive disease)	12 (57.1)	13 (41.9)	0.28
Other neuro-psychiatric symptoms^a^	11 (52.4)	19 (61.3)	0.52
Gluten sensitivity or Celiac disease^a^	2 (9.5)	2 (6.5)	1.0
Father age at birth (years)^b^	36 (30–47)	36 (25–53)	0.70
Mother age at birth (years)^b^	33 (28–41)	34 (20–40)	0.84
Siblings^b^	1 (0–3)	1 (0–2)	0.72
First-born^a^	11 (52.4)	13 (41.9)	0.46
Twins^a^	0 (0.0)	4 (12.9)	0.14
Birth Weight (g)^b^	3,470 (2,640–4,370)	3,100 (700–4,030)	0.38
Birth Length (cm)^b^	50 (41–55)	50 (32–56)	0.76
Birth weight^a^			0.72
SGA (small for gestational age)	2 (9.5)	5 (16.1)	0.69
AGA (adequate for gestational age)	16 (76.2)	23 (74.2)	0.87
LGA (large for gestational age)	3 (14.3)	3 (9.7)	0.68
Apgar 1'^b^	9 (4–10)	9 (3–10)	0.47
Apgar 5'^b^	10 (7–9)	10 (1–10)	0.19
Pregnancy problems^a^	11 (52.4)	11 (35.5)	0.23
Neonatal problems^a^	7 (33.3)	10 (32.3)	0.94
Income bracket classes^a^			0.78
Class 1	2 (9.5)	2 (6.5)	1.0
Class 2	5 (23.8)	9 (29.0)	0.68
Class 3	10 (47.6)	13 (41.9)	0.69
Class 4	2 (9.5)	6 (19.4)	0.45
Class 5	2 (9.5)	1 (3.2)	0.56
Weaning age (months)^b^	6 (4–7)	6 (4–8)	0.67
Weaning problems^a^	0 (0.0)	3 (9.7)	0.26
Allergies^a^	3 (14.3)	8 (25.8)	0.49
Growth problems^a^	0 (0.0)	4 (12.9)	0.14
Neuromotor development problems^a^	16 (76.2)	22 (71.0)	0.68
- Head maintenance (months)^b^	3 (2–8)	3 (1–5)	0.30
- Sitting position (months)^b^	6 (5–10)	6 (4–9)	0.49
- Crawl (months)^b^	9 (8–11)	8 (6–20)	0.23
- Toddle/walk (months)^b^	12 (11–18)	13 (10–24)	0.29
- Babbling (months)^b^	12 (4–20)	12 (7–36)	0.066
School performance problems or support^a^	4 (19.1)	9 (29.0)	0.42

a *Numero (%)—Fisher's exact test;*

b *Median (1°−3°quartile)—Mann-Whitney U test*.

Other reported comorbidities are asthma, craniosynostosis, plagiocephaly, and obesity.

### Ethics Statement

All subjects' parents gave written informed consent in accordance with the Declaration of Helsinki. The protocol was approved by Ethics Committee of Policlinico Umberto I.

### Statistical Analysis

The distribution of the factors under study was evaluated by means of the Shapiro–Wilk test and the indices of asymmetry (skewness) and kurtosis (kurtosis). Since the main factors did not follow a Gaussian distribution, the quantitative variables were described using the medians and the interquartile range (first and third quartiles) and compared by the Mann–Whitney test. Absolute and percentage frequencies were calculated for qualitative variables, and comparison between groups was performed with Chi-square test or Fisher's exact test, when appropriate. *p* < 0.05 (two-tailed test) were considered statistically significant. The data were collected in an Excel database, and the statistical analysis was carried out with the Stata software version 15.1 (StataCorp, College Station, TX).

Limitations of the study: this is a retrospective study with a small population sample, and some patients had neonatal problems. Strengths of the study: this is an innovative study that investigates an unexplored field, with good-quality statistical analysis and no lack of data.

## Results

Since November 2019, 52 children between 7 and 17 years of age (median 7 years), referred to our center and diagnosed with PANS, have been enrolled in this study.

The patients are 41 males (78.9%) and 10 females (21.1%) (male/female ratio, 4.1:1). See Table 2 for the characteristics of the population.

Of the entire cohort, 32.7% presented neonatal problems: prematurity, testicular agenesis, vomiting, bronchodysplasia, sepsis, lower limb tremors, jaundice, fetal distress at birth, respiratory distress, perinatal asphyxia, bandolier cord, breech birth, difficulty in feeding, hypotonia, neonatal hematemesis, gastroesophageal reflux, infant colic, and anemia.

**Figure 3:**
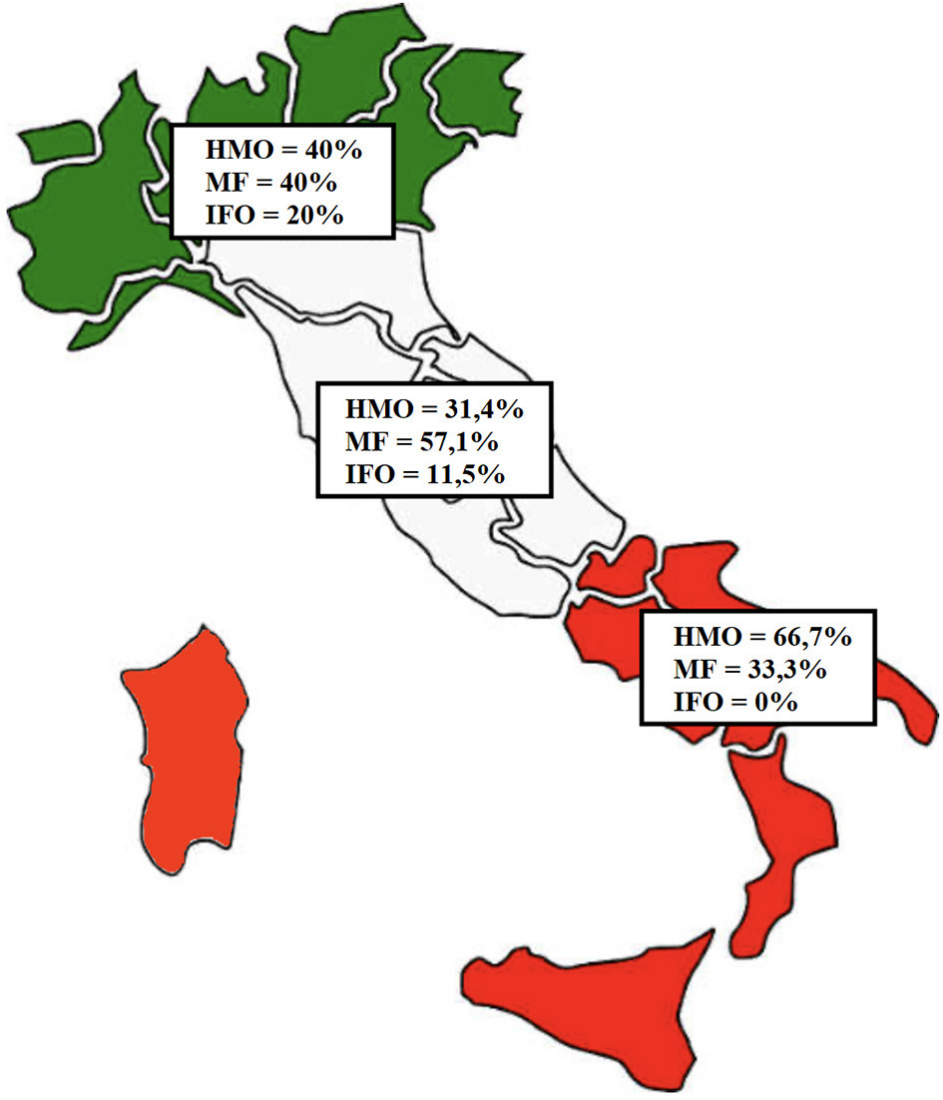
Distribution of the patients based on region of origin. In green, Northern regions (five patients); in white, Central regions (35 patients); in red, Southern regions (12 patients).

By analyzing patient origin, we noticed that 5 (9.6%) children come from the Northern regions of Italy, 35 (67.3%) come from the Central regions, and 12 (23.1%) come from the Southern regions or Italian Islands (Sicily and Sardinia), as shown in Figure 3.

Three groups have been identified among the study population, depending on the kind of feeding they were exposed to during the first 4 months of life: group 1 (21 patients, 40.4%) receiving human milk only (HMO); group 2 (26 patients, 50%) receiving mixed feeding (MF) (i.e., both human milk and infant formula); and group 3 (5 patients, 9.6%) receiving infant formula only (IFO).

Average age at onset is different among the groups: the total is 5.8 years (1.5–10); IFO is earlier, around 4.6 years; HMO is 5.4 years; and MF is older, around 6.3 years.

The IFO group only has five patients, so any statistical analysis and comparison with the other groups appear useless. Therefore, we decided to analyze together the group of patients who received IFO and MF and compare them with the patients who received HMO.

Comparing the two groups in Table 3 [HMO vs. (MF+IFO)], it emerges that the distribution of males is similar in both groups (71.4 vs. 83.9; *p* = 0.32). The age of onset of symptoms is similar in all the groups and ranges from 5 to 6 years.

Breastfeeding is preferred in all regions of Italy, especially in the Southern and Central regions. Food weaning during infant age occurred between 4 and 8 months of life but only three children (5.8%) presented weaning difficulties, and all of them received mixed feeding (11.5% of MF group). Three out of five patients who received only infant formula (60% of the IFO group) had a history of perinatal disease.

The average onset of verbal ability (like babbling and speaking) is around 12 months in both groups but with different age distribution widths, appearing earlier in the HMO group (range 4–20 months) and later in the MF+IFO group (range 7–36 months) (*p* = 0.066).

Although not statistically significant, the following results were observed (see Table 3).

Neonatal problems were found in 32.7% among the overall population, 33.3% among HMO, 26.9% among MF, and 60% among IFO; this may be a study bias, but it is also expected data. Growth problems are present in the MF (11.5%) and IFO (20%) groups but not in HMO. Neurodevelopmental problems are present in HMO (76.2%), MF (69.2%), and IFO (80%).

The time to reach neuro-motor milestones (like head maintenance, reaching sitting position, crawling, and toddling/walking) did not appear to be significantly different among the groups.

Regarding socio-economic status, we analyzed the distribution among family annual income bracket from the poorest (Class 1) to the richest (Class 5) class: 7.7, 26.9, 44.2, 15.4, and 5.8%, respectively (see **Figure 5**). The poorest and richest income brackets prefer exclusive breastfeeding.

Finally, the presence of school performance problems or the need for school support were reported in 13 children (25%): 4 (19.1%) in the HMO group, 6 (23.1%) in the MF group, and 3 (60%) in the IFO group.

School performance problems were reported in 25% of patients. PANS onset occurred in preschool age (<6 years) in 44.2% of cases, and it occurred after the start of school (6 years old or more) in 55.8%. Among the group with preschool onset, only 20.7% presented school performance problems; conversely, it occurred in 30.4% in the group with PANS onset during school age.

These results are not all statistically significant (probably due to the small number of patients), but consistent with our hypothesis since the benefits of fatty acids in the diet are highlighted above all from school age.

## Discussion

The different infant feeding approaches among Italians may be attributed to various reasons that are social, economic, and cultural in nature.

As already specified above, the IFO group only has five patients, so any statistical analysis and comparison with the other groups appear useless. Therefore, we decided to analyze together the group of patients who received IFO and MF, and compare them with the patients who received HMO.

**Figure 4:**
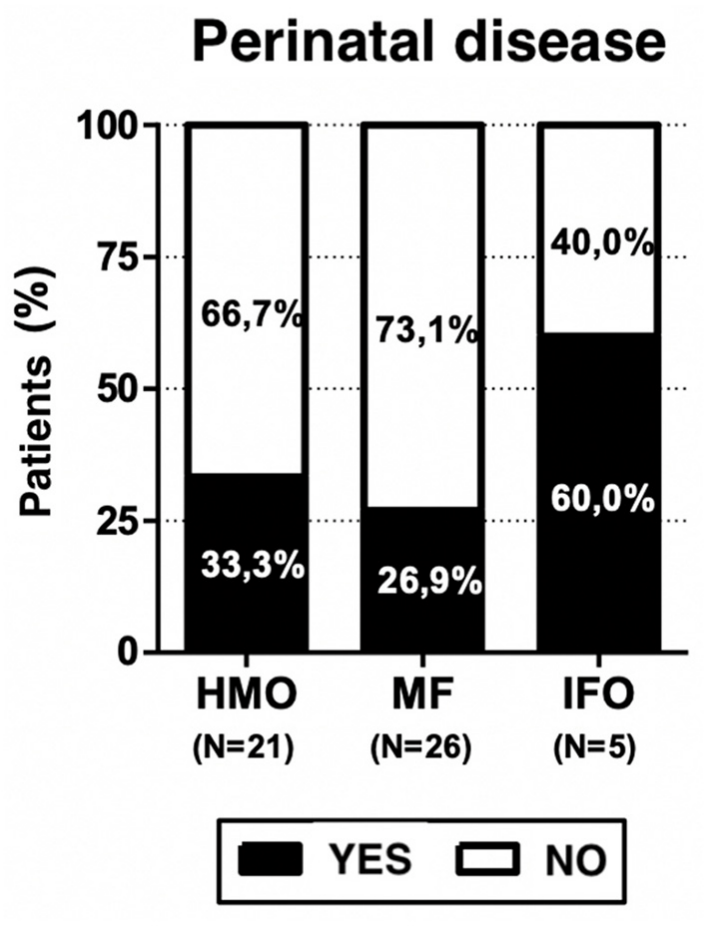
Prevalence of perinatal disease among the study population.

Three out of five patients who received infant formula only (60% of IFO group) had a history of perinatal disease (see Figure 4). On the one hand, it is expected because newborns with perinatal problems often need infant formula; on the other hand, it represents a bias because we have no evidence on how those perinatal problems can affect the neurodevelopment of the children with respect to the other groups, HMO and MF, which presented 33.3 and 26.9% cases, respectively. It is very unlikely that this explains the other main results of the paper because only one (out of five) IFO-fed patient presented with severe Apgar (3–1 min and 1–5 min), bronchodysplasia, and sepsis. One patient presented with lower limb tremors, and one presented with breech position and physiological jaundice, but in any case, the Apgar score was at least 9.

Weaning problems are present only in three patients, all in the MF group (11.5%); these data can be explained with the need to support breastfeeding with infant formula if weaning difficulties occur.

Growth problems are present in the MF and IFO groups but not in HMO; it supports the beneficial activity of breastfeeding and it can be the expression of feeding supplementation needed in children with growth issues.

Neurodevelopmental problems are present in 73.1% of the study population; in detail, HMO, 76.2%; MF, 69.2%; and IFO, 80%. So, the IFO group appears to be more affected; we have to remember that it is the same group with 60% of perinatal problems, and it can represent a confounding factor.

The time to reach neuro-motor milestones (like head maintenance, keeping sitting position, crawling, toddling, or walking) did not appear to be significantly different among the groups.

**Figure 5:**
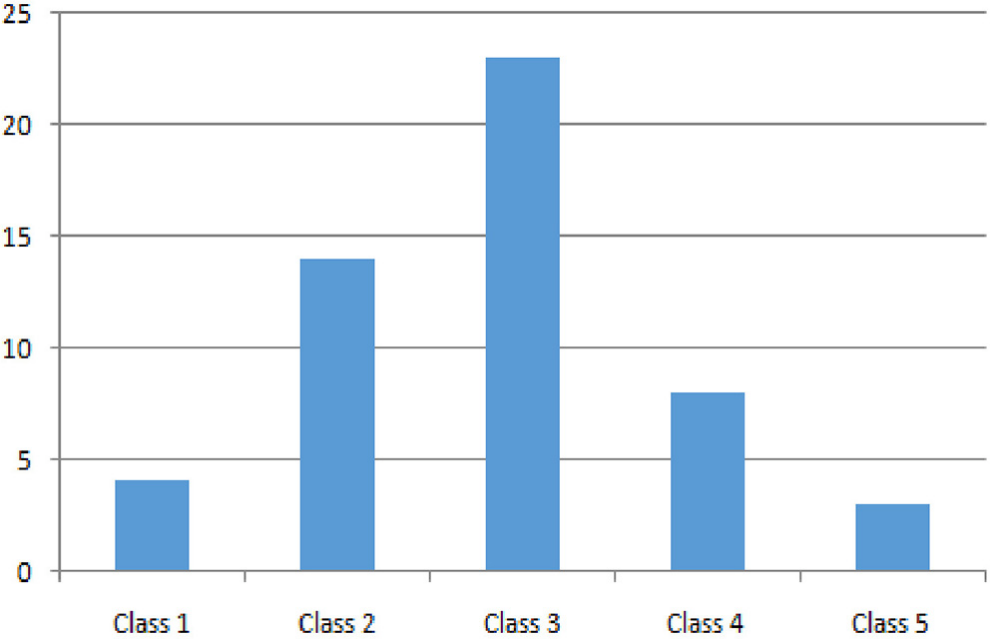
Distribution of patients by income group, from the poorest (Class 1) to the richest (Class 5).

As expected, the distribution among family annual income bracket is pretty normal (see Figure 5), but it is peculiar to notice how the poorest and the richest families prefer exclusive breastfeeding. These data can be explained in many ways: in general, the action of pediatricians during recent decades in family education may have played an important public health role; moreover, the poorest parents probably find adequate and cheap nutrition for their babies in breastfeeding, while the richest ones are probably better instructed.

**Figure 6:**
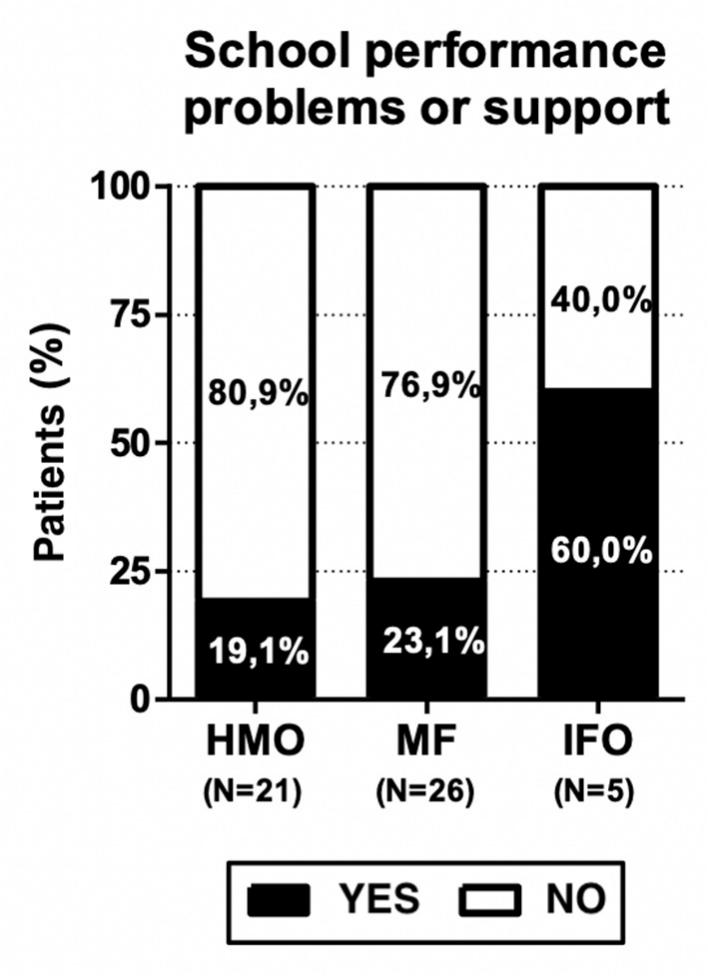
Prevalence of school performance problems among the study population.

The presence of school performance problems and the need for school support were reported in 13 children (25%): 4 (19.1%) patients in the HMO group, 6 (23.1%) in the MF group, and 3 (60%) in the IFO group (see Figure 6). These results are not statistically significant due to the small number of patients, but consistent with our hypothesis since the benefits of fatty acids in the diet are highlighted above all from school age.

The school problems registered in this study are as follows: the need for school support, loss of school years, dysgraphia, dyscalculia, dyslexia, attention deficit, autism spectrum disorder, and oppositional attitude.

## Conclusions

PANS is an underdiagnosed and little known condition among physicians; its incidence is unknown, and this makes it difficult to collect an adequate amount of evidence. Moreover, the follow-up of PANS patients is still short because it is a nosological entity known for a relatively short time and its causes are not that clear.

There are no studies known to the authors that have investigated the role of breastfeeding in the development of PANS. PANS patients have an immunological commitment that can influence the neurological development in children. Breastfeeding has a known immunologic action and a positive influence on the neurodevelopment, so it can be beneficial for PANS children or to prevent PANS onset.

Since the benefits of fatty acids in the diet are evident starting from school age, in line with the working hypothesis, the lack of some fundamental nutrients could play a role in the onset of some educational performance deficits and also of language in its various forms (dysgraphia, dyslexia, dyscalculia, etc.). This lack could also affect the achievement of some milestones in the development of the infant (i.e., babbling). In the group of PANS patients fed only with human milk, we registered fewer cases of growth problems, school performance problems or the need for school support, and a delay in the age of babbling/speaking.

We want to highlight the benefits of breastfeeding in babies and the importance of DHA and all LC-PUFA intake during breastfeeding, since PANS symptoms and PANS diagnosis are typical for those between 3 and 16 years and the benefits of this nutritional elements are evident in adolescence.

Breastfeeding is preferred in all Italian regions, but its diffusion is not homogeneous; it can be explained by social, economic, and cultural phenomena that characterize the different parts of the nation.

The richest and poorest families choose breastfeeding, probably with a different approach and for different reasons (awareness for the former, need for the latter), but this highlights the fact that the health education furnished by pediatricians is precious.

This study offers a food for thought not foreseen *a priori* regarding the topic of breastfeeding in Italy and in Europe, that is, how scientific societies have changed their approach through the years and how it has been received by society and by the various segments of the population.

Promoting breastfeeding is important in the general population as well as in PANS patients because it has an important social and global health impact, also during adult life.

Further studies with a bigger population are needed to investigate the mechanisms underlying PANS and the role that breastfeeding may play in the short- and long-term neurodevelopment of these patients.

## Data Availability Statement

The raw data supporting the conclusions of this article will be made available by the authors, without undue reservation.

## Ethics Statement

The studies involving human participants were reviewed and approved by Policlinico Umberto I, Sapienza University of Rome, Italy. Written informed consent to participate in this study was provided by the participants' legal guardian/next of kin.

## Author Contributions

MM, DB, FD, and AS conceptualized and designed the study, drafted the initial manuscript, and reviewed and revised the manuscript. All authors designed the data collection instruments, collected data, carried out the initial analyses, reviewed and revised the manuscript, conceptualized and designed the study, coordinated and supervised data collection, critically reviewed the manuscript for important intellectual content, and approved the final manuscript as submitted and agree to be accountable for all aspects of the work.

## Conflict of Interest

The authors declare that the research was conducted in the absence of any commercial or financial relationships that could be construed as a potential conflict of interest.

## Abbreviations

PANS: Pediatric acute-onset neuropsychiatric syndrome
PANDAS: Pediatric Autoimmune Neuropsychiatric Disorder Associated with Streptococcal Infections
OCD: Obsessive–compulsive disorders
ENT, ears, nose: and throat
HMO: human milk only
MF: mixed feeding
IFO: infant formula only
ARA: arachidonic acid
WHO: World Health Organization
UNICEF: United Nations International Children's Emergency Fund
LC-PUFA: long-chain polyunsaturated fatty acid
DHA: docosahexanoic acid
S/C: Sieroproteins/Caseins ratio
GOS: galacto-oligosaccharides
FOS: fructo-oligosaccharides
EPA: eicosapentaenoic acid
EU: European Union
IQ: intelligence quotient.
